# Determination of metal(oids) in different traditional flat breads distributed in Isfahan city, Iran: Health risk assessment study by latin hypercube sampling

**DOI:** 10.1016/j.toxrep.2023.02.015

**Published:** 2023-03-06

**Authors:** Seyedeh Mahsa Khodaei, Zahra Esfandiari, Masoud Sami, Ali Ahmadi

**Affiliations:** a Nutrition and Food Security Research Center, Department of Food Science and Technology, School of Nutrition and Food Science, Isfahan University of Medical Sciences, Isfahan, Iran; b Master of Science in Food Hygiene and Safety, Faculty of Veterinary Medicine, Shahrekord Branch, Islamic Azad University, Shahrekord, Iran

**Keywords:** Metal(oid), Bread, Risk assessment, Latin hyper cube sampling, Iran

## Abstract

This survey was conducted to assess the metal(oids) content in 93 samples of bread, including barbari, lavash, and tafton, using inductive couple plasma/optical emission spectroscopy (ICP-OES) method and atomic absorption spectroscopy (AAS). The amounts of measured element were compared with the permissible limit set for bread by FAO/WHO and Iranian National Standardization Organization (INSO). The limit of detection (LOD) was ranged from 6.6 × 10^–5^ to 2.1 × 10^–2^ mg l^−1^ with recoveries ranged from 92% to 102%. The average concentrations of aluminum (Al), arsenic (As), boron (B), cadmium (Cd), iron (Fe), mercury (Hg), magnesium (Mg), sodium (Na), lead (Pb), and zinc (Zn) in bread were 29.88 ± 8, 0.03 ± 0.004, 12.77 ± 3.70, 0.01 ± 0.006, 34.16 ± 8.95, 0.01 ± 0.008, 346.07 ± 36.08, 3314.81 ± 317.19, 0.24 ± 0.11, and 19.65 ± 4.66 mg Kg^−1^, respectively. Amounts of As, Cd, Hg, Mg, Pb, and Zn were lower, and those of Al, Fe, and Na were higher than the permissible limits defined by FAO/WHO. The Latin Hyper Cube (LHC) sampling results revealed that children were exposed to higher non-carcinogenic risk and adults were more threatened by carcinogenic risk. It is recommended to control the entrance of metals in bread in the farm-to-fork chain in order to prevent probable future health challenges.

## Highlights

• The risk assessment of metal(oids) in the most consumed flat breads in Isfahan was carried out.
• TTHQ and TILCR of metal(oids) in bread exceeded the acceptable limits.
• Monitoring and risk assessment of metal(oids) in bread are necessary to done continuously.

## Graphical Abstract

**Figure:**
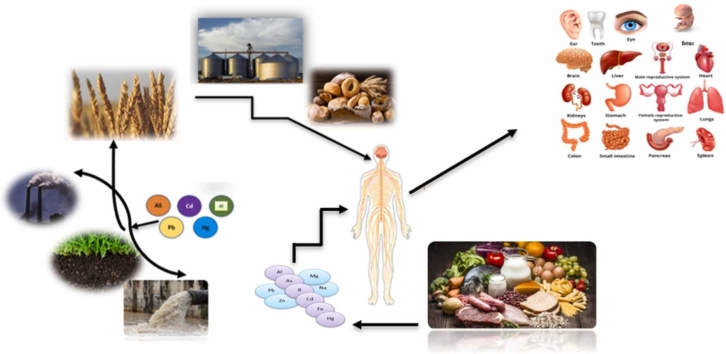

## 1 Introduction

Today, health and safety are one of the main elements for choosing and consuming food. In recent years, food contamination with different elements has become an inevitable challenge [30], [36]. In general, elements are divided into two main groups, essential and non-essential, based on their role in the human body's metabolism. Essential trace elements such as Fe, Zn, copper (Cu), selenium (Se), and manganese (Mn) are effective in human physiological and metabolic activity. However, the consumption of these elements more than the defined limits is harmful to health. Toxic elements such as As, Al, Cd, nickel (Ni), Pb, and Hg are known as unnecessary elements for the body. The consumption of these elements for a long period can adversely effect on human health, even in small amounts [21], [34]. For example, accumulating heavy metals in the human skeleton and fat tissues has side effects and leads to carcinogenic effects, neurological defects, gastrointestinal and respiratory system effects, kidney damage and hepatotoxicity [11], [21], [26], [35]. The usual routes of entering heavy metals into the environment are insecticides, liquid waste, mining, municipal sewage, natural earth crust weathering, and soil erosion. On the other hand, agricultural chemicals such as fertilizers and metal pesticides play a vital role in the contamination of food with trace elements. This problem is particularly prevalent in developing countries where the use of agricultural chemicals is not well controlled [10], [24]. Among various agriculture products, grain-type foods especially wheat are more susceptible to absorbing toxic elements from the environment [30].

Wheat is known as one of the most widely consumed grains around the world and it is used to make products such as flour, bread, sweets, soft drinks, and baby food. Among all these products, bread has a special part in the food basket. Because of its nutritional value, low price, availability, and ease of production, bread is considered the primary and essential part of the diet in many developed and developing countries. Additionally, bread is a good source of vitamins, proteins, nutrients, and fibers [42].

Wheat bread is the most essential and favorite food in the diet of Iranians [17]. A total of 48% of calories and 27% of daily protein of Iranian people are supplied with bread. Traditional Iranian flat breads, including barbari, lavash, and tafton, are prepared with wheat flour and different formulations, shapes, sizes, textures, colors, and flavors [25]. Despite the far-reaching health benefits related to bread consumption, bread contains various heavy metals that can be toxic even in low concentrations [42]. Since heavy metals are known as one of the primary pollutants in the food industry and agricultural products, to prevent the side effect of these types of elements, the levels of them must be maintained within a suitable range, so regulatory organizations in different countries have strictly determined the permissible concentration of these elements in different foodstuffs [29], [33].

In recent years, different provinces of Iran, especially Isfahan had significant growth in industrial activities. Rapid and organized industrial changes, the use of wastewater to irrigate agricultural lands, and numerous steel industries have increased the level of pollution caused by heavy metals in Isfahan province. Today, a large concentration of heavy metals in the cultivated soils of this region and their uptake in cereals, especially wheat, have become a serious human health risk [17]. Therefore, it is necessary to study the risk of exposure to different metals by consuming bread produced in Isfahan bakeries. Estimating the concentration of elements and comparing them with the standard limit in bread has received much attention in the last few decades. Epidemiological evidence and experimental studies have shown that only comparing concentration of toxic metals with standards set by various administrations cannot precisely reflect the side effect of exposure. Quantitative risk assessment of metals is one of the most critical methods to estimate the exposure of carcinogenic and non-carcinogenic risks of elements. Risk assessment findings can provide helpful information for risk managers to make appropriate and effective decisions. In risk analysis, one of the most influential factors is uncertainty calculation, which can be estimated using LHC sampling [2]. A LHC sampling assesses the computing uncertainty of risk factors and study results in generate the probabilities for modelling parameters. The LHC allows acquiring maximum information from uncertain and input quantities, such as exposure factors in order to estimate probabilistic health risk assessment. LHC in the probabilistic risk assessments lessens the uncertainties of the risk calculations with probability distribution for each factor to avoid the overestimation or underestimation [11].

Studies have been shown to evaluate the adverse health risks associated with metal(oids) in food consumed by humans, with some revealing negative health implication while others revealing no negative health effects. For example, the possible risks of exposure to Cd and Pb had been evaluated in rice grains and wheat flour in only one study performed in Isfahan. The results showed that the levels of Cd in sangak and tafton bread were lower than the safe limits set by INSO and Codex Alimentarius Commission. In contrast, Pb level in all mentioned samples, except sangak, was higher than the safe limits. The risk assessment for the result showed that total hazard quotient (THQ) values were higher than one for wheat and Tafton bread, indicating a potential health risk to consumers [17]. In another Iranian study in Golestan province, all examined elements (Cd, Cu, Fe, Pb, and Zn) were within the permissible limits determined by FAO/WHO and INSO and indicating no threat to health of consumers [28]. In the study conducted in Rasht, 40 different types of bread, including barbari, sangak, lavash, and baguette, were analyzed for the presence of some heavy metals (As, Cd, Co, Cr, Hg, Ni, and Pb). It was indicated that high contents of daily intake of Cr, Pb, Cd, and Hg cause undesirable health effects by consumption of bread [27].

Despite the studies done by some researchers, no comprehensive study has been done on the amount of trace elements and its risk assessment in bread samples in Iran, moreover, due to the high consumption of this product in diets of Iranian society, the present study seems essential. Therefore, the aim of this study is firstly, to measure the contents of trace elements (essential and toxic elements) in three commonly consumed bread in Isfahan, Iran, secondly, to evaluate the estimated daily intake (EDI) levels and potential carcinogenic and non-carcinogenic risks associated with the consumption of these types of bread within LHC sampling.

## 2 Materials and methods

### 2.1 Sample collection and preparation

During two months, a total of 93 fresh daily-baked samples of three types of traditional bread (for each 31 samples), including barbari, tafton, and lavash, were randomly obtained from bakeries located in different districts of Isfahan, Iran. Traditional bread is prepared in fermentation process and includes ingredients of flour, water, salt, yeast or sourdough [18]. Each sample was purchased from separated bakery. Therefore, the sampling was performed from 93 bakeries. All samples were kept in polyethylene bags with specific codes until transferring to the laboratory for preparations and analysis.

All the pieces of bread were oven-dried at 50 °C for 48 h to attain constant weight. The dried samples were subjected to the mineralization process through acid digestion to prevent the effect of the organic matrix, the possibility of sample pollution, and losses of analytes. Duplicate weighted samples of each bread were taken into acid-cleaned microwave vessels. A mixture of 8 mL of nitric acid 65%, hydrochloride acid 37%, and hydrogen peroxide 30% was poured into vessels, and kept at room temperature for about 10 min. Then, samples were placed into a microwave digestion system (Anton Paar Muliwave 3000, Germany). After this process, the samples were diluted up to 50 mL in volumetric flasks with deionized water, retained as a stock sample solution, and refrigerated at 4ºC until analysis. All containers used for laboratory analysis were washed with nitric acid 30% and several times with deionized water to prevent secondary contamination in the experiments.

### 2.2 Analysis of metal(oids)

Graphite Furnace Atomic Absorption Spectroscopy (Perkin Elmer Analyst 700, Norwalk, CT, USA) analyzed Pb and Cd in the digested sample solutions. As and Hg were determined by a hydride atomic absorption system. Stock solutions of As, Cd, Hg, and Pb were prepared at 1000 mg l^-1^ concentrations. Standard solutions of each metal were prepared in four concentrations. The concentrations were 40, 10, 40, and 4 ug l^-1^ for Pb, Cd, As, and for Hg [5], [6], [7].

Concentrations of 12 elements (Al, B, Ca, Cr, Cu, Fe, K, Mg, Mn, Na, Se, and Zn) were analyzed in all digested samples by ICP-OES (Varian Vista Pro, Australia). Calibration standards were prepared from a multi-element standard stock solution between 0.1 and 200 mg l^−1^. We used initial calibration blanks (ICB; nitric acid) and initial and continuing calibration verification solutions (ICV and CCV; multi-element standard) for quality assurance. Blanks and ICV solutions were analyzed along with each batch of 10 samples [4]. The wavelengths applied for assessment of the elements amount, according to baseline signals and their interferences at selected lines observed experimentally during the analysis. The LOD is the lowest amount of analyte that can be measured and reliably distinguished from zero but not certainly quantified. Half of LOD was employed in order to evaluate the mean concentration of trace elements when the level was not detected. Method validation parameters for metals analysis were shown in Table 1.

**Table 1:** Parameters for method validation of metals analysis obtained by ICP-OES in bread.

Metals	Wavelength (nm)	r^2^ Value	Recovery%	LOD (mg l^−1^)
Al	308.098	0.995	93	4 × 10^−5^
B	249.77	0.988	96	3.4 × 10^−3^
Ca	396.22	0.996	92	2.1 × 10^−2^
Cr	267.943	0.993	98	1.7 × 10^−5^
Cu	324.047	0.994	99	6.3 × 10^−4^
Fe	239.562	0.996	102	1.6 × 10^−5^
K	766.023	0.991	93	4.5 × 10^−4^
Mg	383.24	0.989	99	5.5 × 10^−5^
Mn	294.089	0.994	96	6.6 × 10^−5^
Na	589.099	0.993	94	4.8 × 10^−4^
Se	206.078	0.991	98	1.5 × 10^−3^
Zn	213.86	0.996	95	2.7 × 10^−4^

### 2.3 Human health risk assessment

Health risk assessment for long-term food based exposure has been introduced as an appropriate tool for identification of risk factors for human health and providing suggestion of risk to management [36]. To evaluate the non-carcinogenic risk, we calculated the probabilistic non-carcinogenic risks associated with the intake of each heavy metal via consuming each bread. In the THQ estimation, it is supposed that the ingestion dose is equal to the adsorbed contaminant dose and that cooking has no effect on the contaminants. THQ is defined as the ratio of the lifetime average daily dose to the oral reference dose (RfD). For this purpose, Eq. 1 was employed to calculate the EDI in mg kg^−1^d^−1^ of each metal of each bread. Then, the THQ for each bread was calculated considering RfD (Eq. 2). TTHQ was obtained by summing up the THQ values of all metals for each bread according to Eq. 3. When THQ or TTHQ was less than one, it shows the population may not involve any adverse health effects. In contrast, if these two parameters were more than one, it presents the most likely experience of adverse health effects for consumers [21], [33], [38]. THQ and TTHQ does not predict the actual adverse health effect on the exposed population but suggests a signal of the risk level due to metal exposure [29].

(1) $$\mathrm{EDI}=\frac{\mathrm{MC}\times \mathrm{IR}\times \mathrm{EF}\times \mathrm{ED}}{\mathrm{BW}\times \mathrm{AT}}$$

(2) $$\mathrm{THQ}=\frac{\mathrm{EDI}}{\mathrm{RFD}}$$

(3) $$\mathrm{TTHQ}=\sum_{i=1}^{n}\mathrm{THQi}$$

Where MC is the concentration of metal (mg kg^−1^ dry weight) in bread samples, IR is the ingestion rate of bread for Iranian adults and children (equivalent to 0.32 kg d^−1^) [25]. EF is the frequency of exposure (365 days per year for both age groups), ED is the duration of exposure to metal(oids) (70 and six years for adults and children, respectively), BW is body weight in kilograms (70 and 15 kg for adults and children, respectively), and AT is the average lifetime, equal to the frequency and duration of metal exposure (25,550 and 2190 days for adults and children, respectively). RfD for Al, As, B, Ca, Cd, Cr, Cu, Fe, Hg, K, Mg, Mn, Na, Pb, Se, and Zn is 0.143, 0.0003, 0.2, 0, 0.001, 1.5, 0.04, 0.3, 0.00071, 0, 0, 0.14, 0.03, 0.004, 0.005, and 0.3 mg kg^−1^ d^−1^, respectively [28].

The EDI values were compared with those mentioned in the INSO and FAO/WHO [14], [19].

Cancer risks related to exposure to a measured dose of a contaminant can be estimated using the incremental lifetime cancer risk (ILCR) (Eq. 4).

(4) ILCR= EDI ×ADAF× CSF

ADAF is the age-dependent adaptation factor, whose value is 1 and 3 for adults and children, respectively. CSF is the cancer slope factor (ug g^−1^ d^−1^) and indicates the probability of oral consumption of a pollutant that increases the risk of carcinogenesis. CSF values for As, Pb, and Cd are 1.5, 0.0085, and 6.3 mg kg^−1^ d^−1^, respectively [38]. The value of this index has not been calculated for Hg and other metals. The present study estimated TILCR due to possible exposure to multiple carcinogenic heavy metals through bread consumption (Eq. 5).

(5) $$\mathrm{TILCR}=\sum_{i=1}^{n}\mathrm{ILCR}$$

The values of ILCR and TILCR are acceptable in the range of 10^−4^ to 10^−6^. Concerning the risk of carcinogenesis, one in a million-cancer risk means that if one million people are exposed to the same concentration of a contaminant over a lifetime, one person would likely contract cancer. If the value of the two indicators is less than 10^−6^, it indicates a slight risk of cancer, and if it is more than 10^−4^, it indicates the risk of cancer due to exposure to the studied traditional bread. In this study, ILCR and TILCR values were compared with the acceptable levels suggested by USEPA [38].

### 2.4 Statistical analysis

The measurements were performed in duplicate and calculated the mean and standard deviation of metal concentrations in Statistical Package for the Social Sciences (SPSS, Inc., USA, version 26). The average concentrations of metal(oids) were compared with INSO and FAO/WHO guidelines [12], [13], [14], [18], [19]. LHC sampling was used with 100,000 repetitions, seed 999, and bin 500 through Crystal Ball software (version 11.1.2.4.600, Oracle, Denver, Co, USA) to estimate THQ, TTHQ, ILCR, and TILCR considering the distribution of independent variables.

## 3 Results and discussion

### 3.1 The concentration of metal(oids)

The amounts of the trace metals in all breads and each type of samples are presented in Table 2, Table 3. With the exception of Ca, Cr, Cu, K, Mn, and Se, which was below the limit of detection, Al, As, B, Cd, Fe, Hg, Mg, Na, Pb and Zn were measured in all samples. Barbari and lavash had the lowest (26.31 ± 7.44 mg kg^−1^) and the highest (34.81 ± 7.89 mg kg^−1^) amounts of Al (Table 3). In addition, the average concentration of Al was 29.88 ± 8 mg kg^−1^ in all bread samples, which is beyond the recommended amount of FAO/ WHO (5 mg kg^−1^). The mean concentration of Al was more than the values reported by Arnich et al., 2012 (2.6 mg kg^−1^), Wang et al., 2020 (4.79 mg kg^−1^) and Ziola-Frankowska et al., 2021 (3.62 mg kg^−1^) [3], [39], [42]. Basaran (2022) reported the average concentration of Al in 30 bread samples from 5.99 ± 0.54 to 117 ± 8.2 mg kg^−1^ [4]. Additionally, high level of Al was presented in steamed bread/bun/cake between 100 and 320 mg kg^−1^ in Hong Kong [41]. The level of As in bread samples ranged from 0.022 to 0.039 mg kg^−1^ (Table 3). The level is below the FAO/ WHO permissible limit (Table 2) and comparable to some records in other studies such as Arnich et al., 2012 (0.025 mg kg^−1^), Ziola-Frankowska et al., 2021 (0.00534 mg kg^−1^), Wang et al., 2020 (0.020 mg kg^-^1), and Basaran, 2022 (0.007–0.056 mg kg^−1^) [3], [4], [39], [42]. The amount of B was from 4.9 to 23.95 mg kg^−1^ and followed the order lavash (10.35 ± 2.67 mg kg^−1^) < barbari (11.84 ± 2.83 mg kg^−1^) < tafton (16.12 ± 2.9 mg kg^−1^) bread (Table 3). The comparison of B concentration is not possible with INSO and WHO/FAO limit because of the lack of limit definition (Table 2). Choi and Jun, 2008 reported the concentration of B in wheat flour (0.181 mg kg^−1^) and Korean white bread (0.12 mg kg^−1^) which was lower than in the present study [9]. In addition, the average concentration of this element in bread and wheat consumed in Istanbul, Turkey, was about 0.7 ± 0.02 mg kg^−1^ [22]. The present study showed that the amounts of Cd in bread (0.01 ± 0.006 mg kg^−1^) was lower than the amount recommended by INSO (0.03 mg kg^−1^) and FAO/ WHO (0.05 mg kg^−1^) (Table 2), indicating no adverse health effect. Cd had almost the same values in all samples but was the lowest among other elements in all bread. In 60 samples of bread examined by Basaran (2022) in Turkey, the concentration of Cd varied from 0.0034 to 0.278 mg kg^−1^[4]. In literature, the average concentration of Cd in different bread types was found to be 0.0193 mg kg^−1^ (Arnich et al., 2012), 0.0482 mg kg^−1^ (Loutfy et al., 2012), 0.024 ± 0.007 mg kg^−1^ (Ghoreishy et al., 2019), and 0.03592 mg kg^−1^ (Ziola-Frankowska et al., 2021) [3], [17], [24], [42]. The amount of Cd varied from 0.02 to 0.03 mg kg^−1^ in different bread produced with wheat flour in Mashhad city in Iran [16]. The reported amount of Cd in Feyzi et al., 2017 (0.00948 ± 0.00475 mg kg^−1^), and Wang et al., 2020 (0.0065 mg kg^−1^) was lower than the amount obtained in our study [15], [39]. Fe concentrations in the different types of bread ranged from 4.83 to 50.88 mg kg^−1^ and followed the order barbari (24.91 ± 4.65 mg kg^−1^) < lavash (34.13 ± 4.87 mg kg^−1^) < tafton (43.43 ± 4.8 mg kg^−1^) (Table 3). The average concentration of Fe in all bread samples of present study was higher and lower than the limits set by FAO/WHO and INSO (Table 2). This situation could be explainable for flour fortification with iron which is the Ministry of Health program in Iran to compensate the iron deficiency. The average concentration of Fe in this study is consistent with the average determined by Feyzi et al., 2017 (30.31 ± 6.8 mg kg^−1^) [15]. However, it is higher than the amount measured by Ziola-Frankowska et al., 2021 (15.13 mg kg^−1^) [42]. Ozbek and Akman, 2016 reported the amount of Fe in white flour bread, whole meal brown bread, whole wheat bread with multi-grains, whole wheat flour bread, and white and whole flour mix varied in a wide range from 15 to 68.7 mg kg^−1^ [32]. Ghasemi et al., 2022 indicated that this element was 43.9 ± 23.57 mg kg^−1^ in bread cooked in a traditional oven, which was more than the values obtained in this study [16]. In the current study, Hg did not differ much in different types of bread, and the average of this element in all bread was estimated to be 0.01 ± 0.008 mg kg^−1^ (Table 2), higher than the study conducted by Arnich et al., 2012 (0.005 mg kg^−1^), Ziola-Frankowska et al., 2021 (0.00263 mg kg^−1^), Feyzi et al., 2017 (0.00438 mg kg^−1^), and Basaran 2022 (0.0009–0.0001 mg kg^−1^) [3], [4], [15], [42]. Compared with FAO/ WHO reference value, Hg doesn’t exceed standard limit, presenting no adverse health effects (Table 2). The mean concentration of Mg in all investigated samples was 346.07 ± 36.08 mg kg^−1^ which is lower than study conducted by Oury et al., 2006 for wheat grain (600–1890 mg kg^−1^) [31]. Additionally, this average is less than defined limit of FAO/ WHO (1000 mg kg^−1^) (Table 2). The average measured amount of Na in all bread samples was 3314.81 ± 317.19 mg kg^−1^ (Table 2), lower than similar investigations in different types of bread from 7000 to 23000 mg kg^−1^ (Carcea et al., 2018), and 7690 ± 2480 mg kg^−1^ (Aalipour et al., 2019) [1], [8]. Na concentration in all and different types of bread exceeded the permissible bread intake level of 1200 mg kg^−1^ set by FAO/ WHO implying short- and long-term adverse effect (Table 2). In our investigation, the average concentration of Pb in all bread samples was 0.24 ± 0.11 mg kg^−1^, less and more than the amount determined by FAO/WHO (2.5 mg kg^−1^) and INSO (0.15 mg kg^−1^), respectively. Ziola-Frankowska et al., 2021, Arnich et al., 2012 and Wang et al., 2020, Basaran, 2022 reported the amount of Pb lower than the current study (0.0385, 0.017, 0.0035, and from 0.002 to 0.115 mg kg^−1^) respectively [3], [4], [39], [42]. In contrast, Loutfy et al., 2012 reported the concentration of Pb higher than the current study (0.33 mg kg^−1^) [24]. Ghoreishy et al., 2019 also estimated that Pb was between 0.15 and 1.32 mg kg^−1^ in wheat bread and rice grains, which was higher than the levels measured in our study [17]. Like Cd, the amount of Pb ist less than FAO/ WHO limit. However, the concentration of Pb exceeded the limit defined by INSO (Table 2). The average amount of Zn was 19.65 ± 4.66 mg kg^−1^ in this study which was lower than the values measured by Ozbek et al., 2016 (10.2–48.6 mg kg^−1^) and higher than the values measured by Feyzi et al., 2017 (14.27 ± 10.10 mg kg^−1^), Ghasemi et al., 2022 (8.74–9.83 mg kg^−1^), and Zioła-Frankowska et al., 2021 (8.89 mg kg^−1^) [15], [16], [32], [42]. The amount of this element is less than FAO/ WHO limit showing no adverse effects on health (Table 2).

**Table 2:** The concentration (mg kg^−1^) of metal(oids) in all examined breads (n = 93).

Type of metal	Minimum	Maximum	Mean ± SD	Reference Value^a^	
				INSO	FAO/ WHO
Al	13.25	57.78	29.88 ± 8	NM^b^	5
As	0.022	0.38	0.03 ± 0.004	NM^b^	0.1
B	4.90	23.95	12.77 ± 3.70	NM^b^	NM^b^
Cd	0.002	0.029	0.01 ± 0.006	0.03	0.05
Fe	4.83	50.88	34.16 ± 8.95	85	5
Hg	0.001	0.032	0.01 ± 0.008	NM^b^	0.03
Mg	291.956	427.23	346.07 ± 36.08	NM^b^	1000
Na	2681.43	4393.2	3314.81 ± 317.19	NM^b^	1200
Pb	0.01	0.46	0.24 ± 0.11	0.15	2.5
Zn	10.28	28.46	19.65 ± 4.66	NM^b^	50

a mg kg^−1^

b Not Mentioned

**Table 3:** The amount (mg kg^−1^) of trace elements in barbari, lavash, and Tafton breads (for each 31 samples).

	Barbari			Lavash			Tafton		
Elements	Min	Max	Mean ± SD	Min	Max	Mean ± SD	Min	Max	Mean ± SD
Al	13.25	45.82	26.31 ± 7.74	20.26	57.78	34.81 ± 7.89	12.23	38.45	28.53 ± 5.84
As	0.022	0.039	0.03 ± 0.004	0.024	0.039	0.03 ± 0.004	0.022	0.039	0.03 ± 0.004
B	4.9	21.57	11.84 ± 2.83	5.51	15.00	10.35 ± 2.67	10.02	23.95	16.12 ± 2.90
Cd	0.002	0.025	0.01 ± 0.008	0.003	0.009	0.007 ± 0.001	0.011	0.027	0.01 ± 0.005
Fe	4.83	33.50	24.91 ± 4.65	22.62	42.42	34.13 ± 4.87	35.29	50.88	43.43 ± 4.80
Hg	0.01	0.03	0.01 ± 0.008	0.01	0.03	0.01 ± 0.007	0.001	0.009	0.007 ± 0.003
Mg	291.95	358.44	326.65 ± 20.45	360.71	427.23	389.54 ± 19.86	293.47	356.9	322.03 ± 15.48
Na	2681.43	3764.6	3267.78 ± 361.67	3000.12	3810.87	3437.19 ± 207.64	2800.79	4393.2	3239.47 ± 333.16
Pb	0.3	0.45	0.36 ± 0.04	0.21	0.31	0.25 ± 0.02	0.04	0.18	0.1 ± 0.04
Zn	11.90	28.46	19.02 ± 4.67	10.28	27.04	17.34 ± 4.36	12.48	31.03	22.59 ± 3.33

Various factors can be the reason for the difference between the results of current and literature values for metal contents. The most influential factor in farm and agriculture is raw wheat grain. Other essential criteria can be the season of wheat harvest and soil composition. In processing, some ingredients, including salt, wheat flour type, the percentage of flour bran, stabilizers, preservatives, and emulsifiers can increase the probability of different metals entrance in bread. The bakery equipment, cooking technique, and the type of fuel may increase metals in the processing line of bread. Therefore, it is necessary to monitor the amounts of metals in traditional bread samples [4], [17], [24], [35], [36].

### 3.2 Estimation of potential human health risks

The toxicity of metal(oids) is influenced by exposure doses, duration and the amount of food consumption. Based on the age of individual to individual, these criterions are different. Evaluation between risks and benefits is assessed by estimating daily consumption [29]. The EDIs obtained in the current study were comparable with the provisional tolerable daily intake (PTDI) (mg.kg^−1^bw per day) defined by joint FAO/WHO food standards [12], [13], [14]. The comparison of EDIs for all of the essential and toxic elements in bread with their PTDI is presented in Table 4. Except for Al (for children group), Na (for both groups of adult and children) and Zn (for children group) which exceeded the limit, all metals are within the PTDI. This situation refers that exposure to these metals has no potential health effects (Table 4).

**Table 4:** Comparison of EDI for studied metals in bread samples in two groups of children and adults with the Provisional Tolerable Daily Intake (PTDI) defined by FAO/ WHO [12], [13], [14].

Element	PTDI^a^	EDI	
		Adult	Children
Al	0.28	0.13	0.63
As	0.002	1.37 × 10^−4^	6.4 × 10^−4^
B	NM^b^	0.05	0.27
Cd	0.008	4.57 × 10^−5^	2.13 × 10^−4^
Fe	0.8	0.15	0.72
Hg	0.0005	4.57 × 10^−5^	2.13 × 10^−4^
Mg	NM^b^	1.58	7.38
Na	0.03	15.15	70.71
Pb	0.003	1.09 × 10^−3^	5.12 × 10^−3^
Zn	0.3	0.08	0.41

a mg.kg^−1^bw per day

b Not Mentioned

**Fig. 1:**
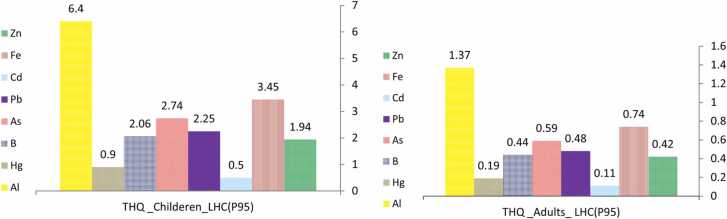
Uncertainty analysis for THQ of investigated metal(oids) in breads (95% confidence limits) for both groups of adults and children.

The THQ of Al, As, B, Fe, Pb (except Tafton bread), and Zn exceeded 1 in children group for three different types of bread (Table 5), while that of Na for all breads exceeded 1 in both age groups. These findings give an indication that children are the most likely to involvement non-carcinogenic health risks from the ingestion of Al, As, B, Fe, Pb, and Zn in three types of bread in long term. Additionally, this condition observes for Na in children and adults. The TTHQ as a criterion for collective effect of multiple metal consumption in all types of bread was surpassed 1. In general, Na plays an essential role in increasing the level of TTHQ in both groups. The results of this study indicated that children are exposed to non-cancer risks 5.73 times more than adults. In calculation of uncertainty of THQ by LHC sampling (Fig. 1), THQ related to Al in adults was greater than one (1.37), while THQ for other elements was less than 1. However, THQ for all metals except Cd and Hg was more than 1 in children group. The THQ values obtained from uncertainty with 95% confidence limits for Na in adults was 35.13, while these values in children was 204.95, respectively (Data not shown). TTHQ results of this study are consistent with those of some similar studies and inconsistent with some others. For example, Pirsaheb et al., 2021 reported that the TTHQ levels of heavy metals in examined cereals were below than the acceptable limits [33]. In contrast, the results of the study conducted by Ghoreishy et al., 2019 showed that THQ for Pb in Tafton and wheat bread was higher than one. Also, the amount of THQ for Pb in Sangak bread prepared in Isfahan was 0.19, and the THQ levels for Cd and Pb in wheat bread were 0.11 and 0.54, respectively [17]. Lei et al., 2015 evaluated THQ in wheat flour of five cities in China. The results showed that the amount of THQ caused by flour consumption was less than one in all age groups of the investigated areas, except for children in Jingyang city [23]. Children were 1.4 times more than adults exposed to non-carcinogenic risks caused by heavy metals in this region. In other study, the amounts of TTHQ in traditional Ethiopian flat bread varied from 6.52 to 8.43. This value indicates considerable potential health effects from the consumption of bread. Al, Mn, and Fe was a major contributor to the TTHQ values [40].

**Table 5:** Estimated Target Hazard Quotient (THQ) in adults and children from metal exposure.

Elements	Adult			Children		
	Barbari	Lavash	Tafton	Barbari	Lavash	Tafton
Al	0.84	0.11	0.91	3.92	5.19	4.25
As	0.45	0.45	0.45	2.13	2.13	2.13
B	0.27	0.23	0.36	1.26	1.10	1.71
Cd	0.04	0.03	0.04	0.21	0.14	0.21
Fe	0.37	0.52	0.66	1.77	2.42	3.08
Hg	0.06	0.06	0.04	0.3	0.3	0.21
Na	29.87	31.42	29.61	174.28	183.31	172.77
Pb	0.41	0.28	0.11	1.92	1.33	0.53
Zn	0.28	0.26	0.34	1.35	1.23	1.60

**Fig. 2:**
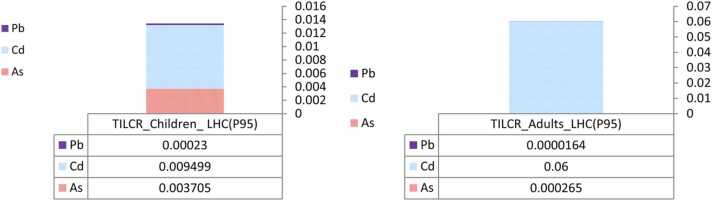
TILCR uncertainty analysis from the consumption of heavy metals in bread in two investigated groups of present study.

The probabilistic approaches for carcinogenic risk assessment in three types of breads (barbari, lavash, and tafton) were presented in Table 6. TILCR for all bread samples in groups adults and children was 2.65E-04 and 3.7E-03 for As, 6.78E-02 and 9.49E-03 for Cd, and 1.64E-05 and 2.3E-04 Pb, respectively (Data not shown). In children and adults, TILCR values obtained from uncertainty analysis was 0.01 and 0.06, respectively (Fig. 2). It means adults are six times more exposed to cancer risks caused by heavy metals than children. USEPA sets a target risk level of more than 10^−4^ threshold to show risk of cancer for individual and all potential carcinogenic contaminants. In this study, TILCR based on the intake of As, Cd, and Pb (except adult group) exceeded this limit for both groups. Hence, it is essential to decrease public health risks by imposing strict rules for all toxic elements, especially in bread to prevent toxic elements contamination. Manufacturers of bread and other health-related foodstuff should guarantee the proper quality of their products by improving their processing technique and choosing raw materials. Compared to the results of other studies, it was shown in Nigeria, the concentrations of Pb and Cd in bread were 0.01–0.071 and 0.01–0.03 mg Kg^−1^, respectively. Besides, Pb and Cd were more and less than the permissible limits set by USEPA and WHO; the ILCR of this bread (3 ×10^−8^) was lower than in our study [37]. The results of the study conducted to measure heavy metals in the commonly consumed cereals of Kermanshah indicated that the levels of TILCR were lower than the values calculated by the uncertainty analysis in the current study [33]. According to the study performed in Bangaladesh, Pb had the highest amount as well as the concentration of As exceeded the permissible limit in wheat. The calculated ILCR for As and Pb due to cereal consumption in children was 0.13 and 0.01, respectively. This amount in adults was 0.07 and 5.5 × 10^−3^ for the mentioned elements in group adult in a study reported by Islam et al., 2015 [20]. Despite the health issues related to the consumption of evaluated bread distributed in Isfahan, we admit that sample size as well as type of bread was small and so it is suggested that further studies be undertaken to approve these findings. Another limitation of this study is the lack of the permissible values and PTDI for some metals in the INSO and FAO/ WHO. Therefore, it was impossible to compare the results of this study with reference values.

**Table 6:** Calculation of incremental lifetime cancer risk (ILCR) of investigated toxic metals in investigated breads of current study, for both age groups of adult and children.

Trace elements	Adult			Children		
	Barbari	Lavash	Tafton	Barbari	Lavash	Tafton
As	2.05 × 10^−4^	2.05 × 10^−4^	2.05 × 10^−4^	2.88 × 10^−3^	2.88 × 10^−3^	2.88 × 10^−3^
Cd	2.88 × 10^−4^	2.01 × 10^−4^	2.88 × 10^−4^	4.03 × 10^−3^	2.82 × 10^−3^	4.03 × 10^−3^
Pb	1.39 × 10^−5^	9.71 × 10^−6^	3.88 × 10^−6^	1.95 × 10^−4^	1.36 × 10^−4^	5.44 × 10^−5^

## 4 Conclusions

In this study, the order of amount of essential and non-essential elements based on their average concentration in bread samples was as follows: Na > Mg > Fe > Al > Zn > B > Pb > As > Hg > Cd. The concentration of some elements such as As, Cd, Hg, Mg, Pb, and Zn was lower than the permissible limits set by FAO/ WHO. Additionally, the concentration of Al, Fe, and Na exceeded the FAO/ WHO limits. The health risk assessment results for children and adults showed that children are exposed to higher non-carcinogenic risk. In contrast, the adults were more threatened by carcinogenic risk compared to children. Al and Na in adults and children were the main causes of exceeding TTHQ. Furthermore, the reason for exceeding TILCR in both age groups was related to existence of As, Cd, and Pb in bread. In the present study, it was examined three types of bread in terms of contamination and health risk status. Adverse effect on health will increase with the consumption of other contaminated food. Hence, the evaluation of metals in the whole diet is recommended. Additionally, it is strongly suggested to perform different control plans in the farm-to-fork chain in agriculture and processing to reduce the concentration of metal(oids). Due to low size of samples, further studies should be performed to support these conclusions.

## Declaration of Competing Interest

The authors declare that they have no known competing financial interests or personal relationships that could have appeared to influence the work reported in this paper.

## Data Availability

The data that has been used is confidential.

## References

[1] Aalipour Hafshajani F., Mahdavi Hafshajani F., Aalipour Hafshajani M. (2019). Evaluation of salt, sodium, and potassium intake through bread consumption in Chaharmahal and Bakhtiari Province. Int. J. Epidem. Res..

[2] Abyani M., Bahaari M.R. (2020). A comparative reliability study of corroded pipelines based on Monte Carlo Simulation and Latin Hypercube Sampling methods. Int. J. Pres. Vess. Pip..

[3] Arnich N., Sirot V., Riviere G., Jean J., Noel L., Guerin T., Leblanc J.C. (2012). Dietary exposure to trace elements and health risk assessment in the 2nd French Total Diet Study. Food Chem. Toxicol..

[4] Basaran B. (2022). Comparison of heavy metal levels and health risk assessment of different bread types marketed in Turkey. J. Food Comp. Anal..

[5] BS. (2002). Determination of trace elements- Determination of mercury by cold-vapor atomic absorption spectrometry (CVAAS) after pressure digestion performed by British Standard. 13806.

[6] BS. (2003). Determination of trace elements. Determination of lead, cadmium, zinc, copper and iron by atomic absorption spectrometry (AAS) after microwave digestion performed by British Standard. 14084.

[7] BS. (2005). Determination of trace elements: Determination of total arsenic and selenium by hydride generation atomic absorption spectrometry (HGAAS) after pressure digestion performed by British Standard. 3.

[8] Carcea M., Narducci V., Turfani V., Aguzzi A. (2018). A survey of sodium chloride content in Italian artisanal and industrial bread. Food.

[9] Choi M.K., Jun Y.S. (2008). Analysis of boron content in frequently consumed foods in Korea. Biol. Trace Elem. Res..

[10] Defarge N., Vendomois J.S.D., Seralini G.E. (2018). Toxicity of formulants and heavy metals in glyphosate-based herbicides and other pesticides. Toxicol. Report..

[11] Eghbaljoo-Gharehgheshlaghi H., Shariatifar N., Arab A., Alizadeh-Sani M., Karimi Sani I., Asdagh A., Rostami M., Alikord M., Arabameri M. (2020). The concentration and probabilistic health risk assessment of trace metals in three type of sesame seeds using ICP- OES in Iran. Int. J. Environ. Anal. Chem..

[12] FAO/ WHO. (2011). Inventory of substances used as processing aids performed by joint FAO/ WHO standards program Codex Committee on food additives.

[13] FAO/ WHO. (2016). Contaminants and toxins performed by the codex committee on contaminants in foods.

[14] FAO/ WHO. (2019). General Standard for contaminants and toxins in food and feed. Cxs193–1995 Adopted in 1995 Revised in 1997, 2006, 2008, 2009 Amended in 2010, 2012, 2013, 2014, 2015, 2016, 2017, 2018, 2019 performed by the codex committee on contaminants in foods.

[15] Feyzi Y., Malekirad A., Fazilati M., Salavati H., Habibollahi S., Rezaei M. (2017). Metals that are important for food safety control of bread Product. Adv. Biores.

[16] Ghasemi S., Hashemi M., Gholian Aval M., Safarian M., Khanzadi S., Orooji A., Tavakoly Sany S.B. (2022). Effect of baking methods types on residues of heavy metals in the different breads produced with wheat flour in Iran: A case study of Mashhad. J. Chem. Health Risks.

[17] Ghoreishy F., Salehi M., Fallahzade J. (2019). Cadmium and lead in rice grains and wheat breads in Isfahan (Iran) and human health risk assessment. Human. Ecol. Risk Assess..

[18] INSO. (2015). Traditional breads: Specification and test methods performed by Iranian National Standardization Organization. No. 2628.

[19] INSO. (2021). Food and Feed: Maximum limit of heavy metals and test methods performed by Iranian National Standardization Organization. No. 12968.

[20] Islam M.S., Ahmed M.K., Al-Mamun M.H., Raknuzzaman M. (2015). The concentration, source and potential human health risk of heavy metals in the commonly consumed foods in Bangladesh. Ecotoxicol. Environ. Saf..

[21] Kiani A., Arabameri M., Moazzen M., Shariatifar N., Aeenehvan S., Jahed Khniki G., Abdel-Wahhab M., Shahsavari S. (2021). Probabilistic health risk assessment of trace elements in baby food and milk powder using ICP-OES Method. Biol. Trace Elem. Res..

[22] Kuru R., Yilmaz S., Tasli P.N., Yarat A., Sahin F. (2019). Boron content of some foods consumed in Istanbul, Turkey. Biol. Trace Elem. Res..

[23] Lei L., Liang D., Yu D., Chen Y., Song W., Li J. (2015). Human health risk assessment of heavy metals in the irrigated area of Jinghui, Shaanxi, China, in terms of wheat flour consumption. Environ. Monit. Assess..

[24] Loutfy N., Mentler A., Shoeab M., Ahmed M.T., Fuerhacker M. (2012). Analysis and exposure assessment of some heavy metals in foodstuffs from Ismailia city, Egypt. Toxicol. Environ. Chem..

[25] Mohammadbeigi A., Salehi A., Izanloo H., Ghorbani Z., Vanaki V., Ramazani R., Asadi-Ghalhari M. (2018). Prevalence of using baking soda in different types of most commonly consumed breads by Iranian people. Adv. Human. Biol..

[26] Munir N., Jahangeer M., Bouyahya A., El Omari N., Ghchime R., Balahbib A., Aboulaghras S., Mahmood Z., Akram M., Ali Shah S.M., Mikolaychik I.N., Derkho M., Rebezov M., Venkidasamy B., Thiruvengadam M., Shariati M.A. (2022). Heavy metal contamination of natural foods ss a serious health issue: A review. Sustain.

[27] Naghipour D., Amouei A., Nazmara S. (2014). A comparative evaluation of heavy metals in the different breads in iran: a case study of Rasht City. Health Scope.

[28] Nejabat M., Kahe H., Shirani K., Ghorbannejad P., Hadizadeh F., Karimi G. (2017). Health risk assessment of heavy metals via dietary intake of wheat in Golestan Province, Iran. Human. Ecol. Risk Assess. Inter. J..

[29] Nyarko E., Boateng C.M., Asamoah O., Edusei M.O., Mahu E. (2023). Potential human health risks associated with ingestion of heavy metals through fish consumption in the Gulf of Guinea. Toxicol. Rep..

[30] Orisakwe O.E., Kanayochukwu Nduka J., Nwadiuto Amadi C., Onyekachi Dike D., Bede O. (2012). Heavy metals health risk assessment for population via consumption of food crops and fruits in Owerri, South Eastern, Nigeria. Chem. Cent. J..

[31] Oury F.X., Leenhardt F., Remesy C., Chanliaud E., Duperrier B., Balfourier F., Charmet G. (2006). Genetic variability and stability of grain magnesium, zinc and iron concentrations in bread wheat. Eur. J. Agron..

[32] Ozbek N., Akman S. (2016). Method development for the determination of calcium, copper, magnesium, manganese, iron, potassium, phosphorus and zinc in different types of breads by microwave induced plasma-atomic emission spectrometry. Food Chem..

[33] Pirsaheb M., Hadei M., Sharafi K. (2021). Human health risk assessment by Monte Carlo simulation method for heavy metals of commonly consumed cereals in Iran- Uncertainty and sensitivity analysis. J. Food Compos. Anal..

[34] Sharafi K., Yunesian M., Nabizadeh Nodehi R., Mahvia A.H., Pirsaheb M. (2019). A systematic literature review for some toxic metals in widely consumed rice types (domestic and imported) in Iran: Human health risk assessment, uncertainty and sensitivity analysis. Ecotoxicol. Environ. Saf..

[35] Shariatifar N., Mozaffari Nejad A.S., Ebadi Fathabad A. (2017). Assessment of heavy metal content in refined and unrefined salts obtained from Urmia, Iran. Toxin Rev..

[36] Shariatifar N., Seilani F., Jannat B., Nazmara S., Arabameri M. (2020). The concentration and health risk assessment of trace elements in commercial soft drinks from Iran maketed. Int. J. Environ. Anal. Chem..

[37] Udowelle N.A., Igweze Z.N., Asomugha R.N., Orisakwe O.E. (2017). Health risk assessment and dietary exposure of polycyclic aromatic hydrocarbons (PAHs), lead and cadmium from bread consumed in Nigeria. Rocz. Panstw. Zakl. Hig..

[38] USEPA, U. (2011). Integrated Risk Information System Assessments performed by United States Environmental Protection Agency.

[39] Wang B., Liu Y., Wang H., Cui L., Zhang Z., Guo J., Liu S., Cui W. (2020). Contamination and health risk assessment of lead, arsenic, cadmium, and aluminum from a total diet study of Jilin Province, China. Food Sci. Nutr..

[40] Woldetsadik D., Llorent-Martínez E.J., Ortega-Barrales P., Haile A., Hailu H., Madani N., Warner N.S., Fleming D.E.B. (2020). Contents of metal(loid)s in a traditional ethiopian flat bread (Injera), dietary intake, and health risk assessment in addis Ababa, Ethiopia. Biol. Trace Elem. Res..

[41] Wong W.W.K., Chung S.W.C., Kwong K.P., Ho Y.Y., Xiao Y. (2010). Dietary exposure to aluminum of the Hong Kong population. Food Add. Cont..

[42] Ziola-Frankowska A., Karas K., Mikolajczak K., Kurzyca I., Kowalski A., Frankowski M. (2021). Identification of metal(loid)s compounds in fresh and pre-baked bread with evaluation of risk health assessment. J. Cereal. Sci..

